# Supplementary material for: Pyruvate Dehydrogenase Kinase 1 inhibition mediated oxidative phosphorylation enhancement in cartilage promotes osteoarthritis progression
Source: BMC Musculoskelet Disord. 2023 Jul 20;24:597. doi: 10.1186/s12891-023-06585-6 (PMC10357736; doi:10.1186/s12891-023-06585-6)
Supplement: Supplementary file 1 — Additional file 1. [file 12891_2023_6585_MOESM1_ESM.zip › BP.Gsea.1653623667859/GOBP_LYMPH_VESSEL_DEVELOPMENT.html]

Details for gene set GOBP\_LYMPH\_VESSEL\_DEVELOPMENT[GSEA]

|  || Dataset | 55457\_collapsed\_to\_symbols.cls.cls #OA\_versus\_normal.cls.cls #OA\_versus\_normal\_repos |
| Phenotype | cls.cls#OA\_versus\_normal\_repos |
| Upregulated in class | normal |
| GeneSet | GOBP\_LYMPH\_VESSEL\_DEVELOPMENT |
| Enrichment Score (ES) | -0.52591926 |
| Normalized Enrichment Score (NES) | -1.7197429 |
| Nominal p-value | 0.0053097345 |
| FDR q-value | 0.120484374 |
| FWER p-Value | 1.0 |
Table: GSEA Results Summary

  

Fig 1: Enrichment plot: GOBP\_LYMPH\_VESSEL\_DEVELOPMENT      
 Profile of the Running ES Score & Positions of GeneSet Members on the Rank Ordered List

  

| SYMBOL | TITLE | RANK IN GENE LIST | RANK METRIC SCORE | RUNNING ES | CORE ENRICHMENT || 1 | VEGFC | vascular endothelial growth factor C [Source:HGNC Symbol;Acc:HGNC:12682] | 2239 | 0.335 | -0.1318 | No |
| 2 | SYK | spleen associated tyrosine kinase [Source:HGNC Symbol;Acc:HGNC:11491] | 4084 | 0.147 | -0.2565 | No |
| 3 | HEG1 | heart development protein with EGF like domains 1 [Source:HGNC Symbol;Acc:HGNC:29227] | 4117 | 0.144 | -0.2409 | No |
| 4 | PROX1 | prospero homeobox 1 [Source:HGNC Symbol;Acc:HGNC:9459] | 4299 | 0.128 | -0.2388 | No |
| 5 | TBX1 | T-box transcription factor 1 [Source:HGNC Symbol;Acc:HGNC:11592] | 4679 | 0.098 | -0.2560 | No |
| 6 | FOXC1 | forkhead box C1 [Source:HGNC Symbol;Acc:HGNC:3800] | 5227 | 0.054 | -0.2917 | No |
| 7 | TMEM204 | transmembrane protein 204 [Source:HGNC Symbol;Acc:HGNC:14158] | 5229 | 0.054 | -0.2850 | No |
| 8 | PPP3CB | protein phosphatase 3 catalytic subunit beta [Source:HGNC Symbol;Acc:HGNC:9315] | 5311 | 0.046 | -0.2854 | No |
| 9 | PDPN | podoplanin [Source:HGNC Symbol;Acc:HGNC:29602] | 5594 | 0.024 | -0.3043 | No |
| 10 | SOX18 | SRY-box transcription factor 18 [Source:HGNC Symbol;Acc:HGNC:11194] | 6053 | -0.008 | -0.3388 | No |
| 11 | SVEP1 | "sushi, von Willebrand factor type A, EGF and pentraxin domain containing 1 [Source:HGNC Symbol;Acc:HGNC:15985]" | 8431 | -0.188 | -0.4998 | Yes |
| 12 | VASH1 | vasohibin 1 [Source:HGNC Symbol;Acc:HGNC:19964] | 8473 | -0.191 | -0.4789 | Yes |
| 13 | PKD1 | "polycystin 1, transient receptor potential channel interacting [Source:HGNC Symbol;Acc:HGNC:9008]" | 8755 | -0.213 | -0.4740 | Yes |
| 14 | LGALS8 | galectin 8 [Source:HGNC Symbol;Acc:HGNC:6569] | 9424 | -0.266 | -0.4924 | Yes |
| 15 | TIE1 | tyrosine kinase with immunoglobulin like and EGF like domains 1 [Source:HGNC Symbol;Acc:HGNC:11809] | 9548 | -0.278 | -0.4671 | Yes |
| 16 | NR2F2 | nuclear receptor subfamily 2 group F member 2 [Source:HGNC Symbol;Acc:HGNC:7976] | 10129 | -0.334 | -0.4701 | Yes |
| 17 | EPHA2 | EPH receptor A2 [Source:HGNC Symbol;Acc:HGNC:3386] | 10219 | -0.343 | -0.4340 | Yes |
| 18 | EFNB2 | ephrin B2 [Source:HGNC Symbol;Acc:HGNC:3227] | 10298 | -0.350 | -0.3960 | Yes |
| 19 | ACVR2B | activin A receptor type 2B [Source:HGNC Symbol;Acc:HGNC:174] | 11011 | -0.431 | -0.3971 | Yes |
| 20 | BMPR2 | bone morphogenetic protein receptor type 2 [Source:HGNC Symbol;Acc:HGNC:1078] | 11174 | -0.454 | -0.3527 | Yes |
| 21 | KDR | kinase insert domain receptor [Source:HGNC Symbol;Acc:HGNC:6307] | 11454 | -0.496 | -0.3120 | Yes |
| 22 | PTPN14 | protein tyrosine phosphatase non-receptor type 14 [Source:HGNC Symbol;Acc:HGNC:9647] | 11797 | -0.548 | -0.2696 | Yes |
| 23 | FOXC2 | forkhead box C2 [Source:HGNC Symbol;Acc:HGNC:3801] | 12218 | -0.650 | -0.2205 | Yes |
| 24 | ACVRL1 | activin A receptor like type 1 [Source:HGNC Symbol;Acc:HGNC:175] | 12242 | -0.656 | -0.1398 | Yes |
| 25 | FLT4 | fms related receptor tyrosine kinase 4 [Source:HGNC Symbol;Acc:HGNC:3767] | 12396 | -0.705 | -0.0631 | Yes |
| 26 | VEGFA | vascular endothelial growth factor A [Source:HGNC Symbol;Acc:HGNC:12680] | 12629 | -0.813 | 0.0210 | Yes |
Table: GSEA details [plain text format]

  

Fig 2: GOBP\_LYMPH\_VESSEL\_DEVELOPMENT      
 Blue-Pink O' Gram in the Space of the Analyzed GeneSet

  

Fig 3: GOBP\_LYMPH\_VESSEL\_DEVELOPMENT: Random ES distribution      
 Gene set null distribution of ES for **GOBP\_LYMPH\_VESSEL\_DEVELOPMENT**
